# The Contribution of Coevolving Residues to the Stability of KDO8P Synthase

**DOI:** 10.1371/journal.pone.0017459

**Published:** 2011-03-09

**Authors:** Sharon H. Ackerman, Domenico L. Gatti

**Affiliations:** 1 Department of Biochemistry and Molecular Biology, Wayne State University School of Medicine, Detroit, Michigan, United States of America; 2 Cardiovascular Research Institute, Wayne State University School of Medicine, Detroit, Michigan, United States of America; The Scripps Research Institute, United States of America

## Abstract

**Background:**

The evolutionary tree of 3-deoxy-*D*-manno-octulosonate 8-phosphate (KDO8P) synthase (KDO8PS), a bacterial enzyme that catalyzes a key step in the biosynthesis of bacterial endotoxin, is evenly divided between metal and non-metal forms, both having similar structures, but diverging in various degrees in amino acid sequence. Mutagenesis, crystallographic and computational studies have established that only a few residues determine whether or not KDO8PS requires a metal for function. The remaining divergence in the amino acid sequence of KDO8PSs is apparently unrelated to the underlying catalytic mechanism.

**Methodology/Principal Findings:**

The multiple alignment of all known KDO8PS sequences reveals that several residue pairs coevolved, an indication of their possible linkage to a structural constraint. In this study we investigated by computational means the contribution of coevolving residues to the stability of KDO8PS. We found that about 1/4 of all strongly coevolving pairs probably originated from cycles of mutation (decreasing stability) and suppression (restoring it), while the remaining pairs are best explained by a succession of neutral or nearly neutral covarions.

**Conclusions/Significance:**

Both sequence conservation and coevolution are involved in the preservation of the core structure of KDO8PS, but the contribution of coevolving residues is, in proportion, smaller. This is because small stability gains or losses associated with selection of certain residues in some regions of the stability landscape of KDO8PS are easily offset by a large number of possible changes in other regions. While this effect increases the tolerance of KDO8PS to deleterious mutations, it also decreases the probability that specific pairs of residues could have a strong contribution to the thermodynamic stability of the protein.

## Introduction

3-Deoxy-*D*-manno-octulosonate 8-phosphate (KDO8P) synthase (KDO8PS) is a bacterial enzyme that synthesizes KDO8P from phosphoenolpyruvate (PEP) and arabinose 5-phosphate (A5P). This reaction is of significant biological relevance, as KDO8P is the phosphorylated precursor of KDO, which is an essential component of the endotoxin of Gram negative bacteria [1]. The enzyme exists in two forms, differing in the requirement (or lack thereof) of a divalent metal for activity [2]. We and others have determined high-resolution structures of both metallo- and non-metallo forms of KDO8PS [2], [3], [4], [5], [6], which revealed the features of the active site: in metal dependent KDO8PSs the divalent metal ion (Zn^2+^, or Fe^2+^, [7]) is coordinated by the side chains of a cysteine, a histidine, a glutamic acid, and an aspartic acid. In non-metallo KDO8PSs an asparagine replaces the metal binding cysteine and there is no metal ion. Several mutagenesis studies [6], [8], [9], [10], [11], [12] and more recently, quantum mechanical simulations [7], [13], [14], have established that substitution of the metal binding cysteine with asparagine is absolutely required to convert metal-dependent to metal-independent KDO8PSs, although additional changes in the highly conserved CysAspGlyPro motif of the loop that contains the metal binding aspartic acid are necessary to achieve appreciable levels of activity [6], [12]. These observations are intriguing and raise the possibility that during the evolution of KDO8PS, after the initial choice between cysteine + metal and asparagine, additional sequence divergence occurred primarily to maximize the stability of the protein in the environment of hundreds of different bacteria. In this study we aimed to establish if the climb to stability fitness was a key factor in determining the amino acid sequence of KDO8PSs, and whether it affected only conserved positions or also positions with a high propensity for coevolution. A new combination of tools from information theory and structural modeling provided an avenue to quantify the contribution of coevolving residues to the stability of KDO8P synthase: this methodology may be of general value in the study of all protein families.

## Results

### The stability landscape of KDO8PS

From the point of view of protein stability the organization of enzyme active sites is inherently unstable because these sites are optimized for catalysis, which means they are pre-organized to stabilize the transition state(s), rather than the protein [15], [16]. Thus, the substitution of a catalytic side chain (most often to alanine) will typically increase the overall protein stability, while sacrificing function [17], [18]. Conversely, most mutations that introduce a new function are destabilizing [19], [20]. The generality of this stability-function tradeoff must be viewed within the context of the fact that regardless of their effect on functions most mutations are destabilizing [21], [22], [23], [24]. With respect to KDO8PS, several attempts were made (e.g., [6], [11]) to map the evolutionary paths from metallo- to non-metallo forms (and vice versa) without giving sufficient consideration to the fact that sequence differences between and within these forms in different bacterial backgrounds, may be related not only to activity but also to stability.

**Figure 1:**
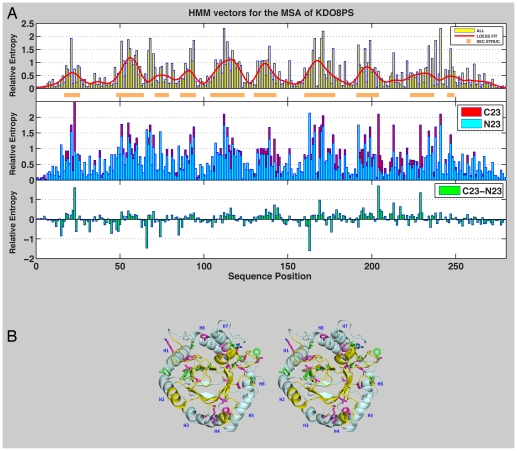
HMM histograms of metallo and non-metallo KDO8PSs. A. 1^st^ inset, HMM vector for all KDO8PSs with superimposed Loess fit (calculated with a span of 20 residues). Regions of the histogram higher than the mean are highlighted with an orange bar below the sequence. 2^nd^ inset, HMM vectors for the C23 and N23 sub-families of KDO8PS. 3^rd^ inset, difference between the HMM vectors of the C23 and N23 sub-families. B. Stereo view of a monomer of *Nm.* KDO8PS shown here as a ribbon drawing. The regions of the structure corresponding to the orange bars in the 1^st^ inset of Panel A are colored in yellow. Residues corresponding to positions with values >2σ in the difference histogram of panel A are shown as sticks with green carbon atoms. Residues corresponding to positions with values >2σ for the difference between C23 and N23 KDO8PSs in the correlation between stability and amino acid usage (last inset in Figure 2B) are shown as sticks with magenta carbon atoms. Helices are labeled (H1 to H8) according to their position in the amino acid sequence.

One way we looked at the relevance of individual residues in the context of the three-dimensional structure of KDO8PSs was by computing a Hidden Markov Model (HMM) for the entire KDO8PS protein family (multiple sequence alignment MSA S1, Supporting Information). This family contains the ‘C23’ metallo sub-family (175 sequences with Cys at position 23 of *Neisseria meningitidis* (*Nm.*) KDO8PS (Uniref Q9JZ55, PDB 2QKF), used here as reference [6]), and the ‘N23’ non-metallo sub-family (173 sequences with Asn at position 23). Only the positions of the MSA corresponding to the sequence of *Nm.* KDO8PS (MSA S2, Supporting Information) were used in the calculation of the HMM profile. A HMM profile specifies a probability distribution over the alphabet of the 20 common amino acids, taking into consideration the background frequency for each amino acid, computed by counting amino acid occurrences in all known proteins, or only in the proteins of the family under consideration [25],[26]. If the background frequency of amino acid *j* is π*_j_*, then the important positions are those whose distribution differs from π. Therefore, the relative entropy between the observed distribution *P*_i_ at the *i*-th position of the profile and π, *H*(*P*_i_ || π) = *I*(*P*_i_), defines the information content of *P*_i_. This information can be visualized as a HMM histogram or vector (Figure 1A). In practice, the height (relative entropy  =  information content) of the histogram at each position indicates how much the observed frequency of amino acids at that position deviates from the background frequency. In the HMM vector of Figure 1A, position numbers refer to the reference sequence of *Nm.* KDO8PS [6]. There is a “wavy” pattern in the histogram derived from the MSA of all KDO8PSs (1^st^ inset of Figure 1A), which is clearly evident in the superimposed Loess [27], [28] fit. Regions of the histogram higher than the mean value are marked with an orange bar below the sequence; these regions invariably include the strands of the β-barrel in the structure (Figure 1B), a clear indication that the sequence of the interior of the protein is less determined by chance. The HMM vectors of metallo (C23) or non-metallo (N23) KDO8PSs, calculated independently, are shown in the 2^nd^ inset of Figure 1A. Their difference is shown in the 3^rd^ inset of Figure 1A. The difference vector identifies positions that best fulfill the specific demands of the two subfamilies of KDO8PS: while partly still in regions of the HMM profile with high relative entropy, these positions tend to cluster outside the β-barrel (Figure 1B).

Unfortunately the HMM vectors do not reveal whether the driving force for these effects is only function or whether stability is also a factor. This question was addressed by using the experimentally validated FoldX algorithm [22], [23], [29] to calculate the ΔΔG changes associated with introducing any one of the 20 possible amino acids at each position of the structure of *Nm.* KDO8PS (again taken here as the reference structure for this class of proteins). This type of calculation was initially introduced by Tokuriki *et al.* [30] to study the overall distribution of stability effects for all possible mutations in a large set of different single domain globular proteins. In our case we used an entire tetramer of *Nm.* KDO8PS, which is known to be the biological unit of the enzyme [6], and the individual mutations were introduced simultaneously in all four subunits. Thus, the calculated ΔΔG changes account also for the effects of mutations at the interface between subunits. Furthermore, as ΔΔG changes can be dependent on a particular conformation of the enzyme, a three-dimensional model of tetrameric *Nm.* KDO8PS derived from the X-ray structure [6] (see Methods section) was subjected to 12 nanoseconds (ns) of molecular dynamics (MD) simulation at 300 K under solvated conditions. This very long simulation time progressively eliminated possible errors in the original model and assured that the equilibrium structure of *Nm.* KDO8PS that is used in the FoldX calculations is as close as possible to the native structure in solution. Progressive convergence of the structure toward equilibrium was evaluated from the changes in the Cα root mean square deviation, Cα-RMSD, from the structure at time *t* = 0 (Figure S1A, Supporting Information). The final part of the simulation (6–12 ns) was selected to gather statistics about the conformational properties of the enzyme. The fluctuations around the average structure (Cα root mean square fluctuation, Cα-RMSF) that occur in this part of the simulation reflect the degree of mobility in the solution structure (Figure S1A).

**Figure 2:**
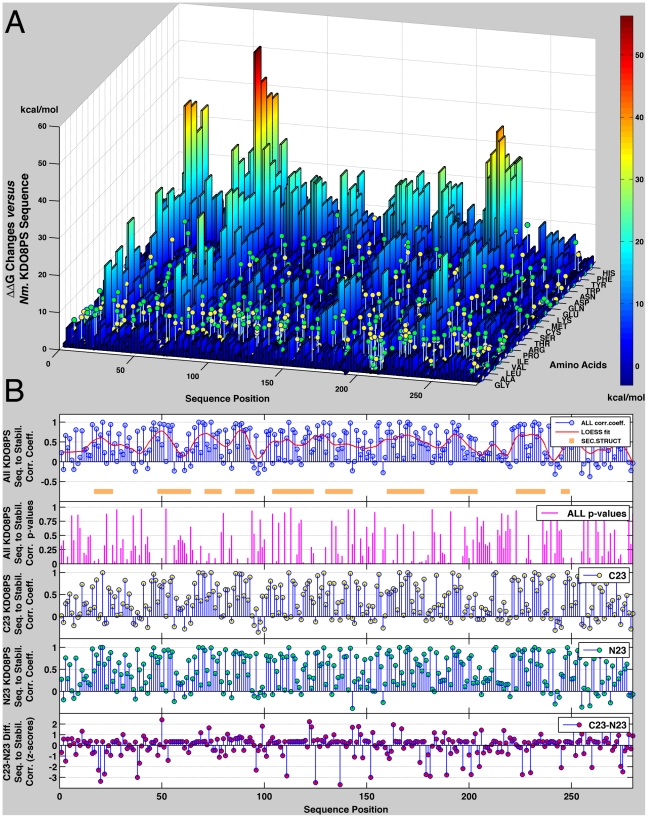
Stability landscape of KDO8PS. A. ΔΔG changes associated with introducing any one of the 20 possible amino acids at each position of all four subunits of the structure of *Nm.* KDO8PS were calculated using the FoldX algorithm to produce a “stability landscape” of KDO8PS: the peaks in the landscape represent positions in the protein where introduction of a certain amino acid would significantly decrease the overall stability. The observed frequency of different amino acids at each position in the two subfamilies of C23 and N23 KDO8PSs is superimposed to the stability landscape as lolly-pops (yellow for C23, green for N23), whose height is proportional to the frequency of a certain amino acid at a certain position. B. Stability/(amino acid usage) correlation in the two subfamilies of KDO8PSs. Correlation coefficients are based on the stability landscape and the relative frequency of each amino acid at each position as shown in panel A. From top to bottom: 1^st^ inset, correlation between stability and amino acid usage at each position in the entire family of KDO8PSs; a Loess fit (calculated with a span of 20 residues) is superimposed. The regions of the structure with the highest relative entropy in the HMM vector of Figure 1A are shown with an orange bar below the stem plot. 2^nd^ inset, *p*-values for the correlations shown in the 1^st^ inset. 3^rd^ and 4^th^ insets, correlation between stability and amino acid usage at each position of C23 (yellow circles) and N23 (green circles) KDO8PSs. 5^th^ inset, difference between the correlations in C23 and N23 KDO8PSs. Residues corresponding to positions with values >2σ for this difference are shown as sticks with magenta carbon atoms in Figure 1B.

Frames from the final 6 ns of the simulation were investigated further with a clustering procedure (incorporated in the program X-Cluster, Schrodinger, LLC) that, based on the cross-RMSDs between frames, identified three structures (PDB S1, PDB S2, PDB S3, Supporting Information) as representative of all the states sampled during this part of the MD run. Thus, the final 6 ns of the MD simulation can be considered as fluctuations around these three main conformations, which appear in a 58∶24∶18 relative ratio. ΔΔG changes associated with mutating every amino acid of all four subunits to all 20 possible amino acids were calculated in duplicate for each of the three conformers. Values derived from each configuration were then merged by weighing each configuration according to its contribution to the population of states in solution as determined from the MD run (Figure S1B). The final ΔΔG values reflect not only the distribution of energies in the solution ensemble of tetrameric *Nm.* KDO8PS, but also the differences originating from the slightly different environment that each residue senses in the four subunits of the tetramer. The outcome of this calculation is a “stability landscape” of KDO8PS (Figure 2A; Energy Matrix S1, Supporting Information): the peaks in the landscape represent positions in the protein where introduction of a certain amino acid would significantly increase the ΔG, and therefore decrease the overall stability. It is worth noting that while the energies derived from FoldX are clearly not on an absolute scale [30], the relative trends are expected to be correct [31], [32]. In general, it can be seen how bulky aromatic residues (W,Y,F,H) tend to decrease stability ( = increase energy) at every position, and in four positions (21,68,231,232) any residue besides glycine or alanine decreases stability dramatically. These effects appear to be due to very large energy terms derived from van der Waals clashes of these residues with their surroundings, which are not sufficiently relieved by the relaxation of the structure. The observed amino acid frequencies at each position in the C23 and N23 sub-families of KDO8PSs are shown as lolly-pops (yellow for C23, green for N23, height proportional to the corresponding frequency) superimposed to the stability landscape of KDO8PS (Figure 2A). In most positions, the amino acids most often used are those that do not decrease stability: in other words, only the planes of the stability landscape are significantly populated.

The data presented in Figure 2A offer an interesting opportunity to understand the relationship between sequence and stability in KDO8PS: for each position in the reference structure of *Nm.* KDO8PS we consider two vectors (each with 20 elements): the first is the vector of the ΔΔG changes associated with mutating the original sequence to each of the 20 amino acids; the other contains the frequencies of each of the 20 amino acids for that position in each of the two subfamilies of KDO8PS (or in the entire family). We recall that if a MSA is composed of independent sequences all producing stable folds with approximately the same structure, and if individual residues contribute additively to stability (no epistasis) [33], [34], then the stability contribution ΔΔG*_a,i_* of a particular amino acid *a* at a given position *i* should be a roughly logarithmic function of its frequency *f_a,i_* in the MSA [35]:
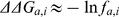
(1)


Approaches based on this idea have been generally successful in engineering more stable proteins [36], [37], [38], [39]. It follows that for each of the 280 positions in the reference structure of *Nm.* KDO8PS we can calculate the linear correlation coefficient (*corr*) between the stability vector [exp(-ΔΔG*_a,i_*)] derived with FoldX and the amino acid frequency vector derived from the MSA. The result of this calculation is shown in the insets of Figure 2B; in the family of all KDO8PSs and in both subfamilies there is a clear distinction between positions in which the amino acid usage is influenced by an evolutionary drive to increase stability, and other positions in which there is no such trend. There is a wavy pattern in the correlation between stability and observed amino acid frequencies (see the Loess fit in the first inset of Figure 2B), which is similar to that already noticed in the HMM vector (Figure 1A). The 2^nd^ inset of Figure 2B shows the *p*-values for the correlations in the 1^st^ inset, calculated by testing the *null* hypothesis of zero correlation against the *alternative* hypothesis of non-zero correlation. The large *p*-values for all the sequence positions with correlation near zero, and the small *p*-values (<5E-4) for all the sequence positions with large correlation, lend credibility to the apparent correlation between stability gains and choice of particular amino acids in certain regions of the protein. The correlation coefficients between the HMM vectors (Figure 1A) and the stability/sequence correlation vectors are 0.34 (*p* = 7.1E-9), 0.31 (*p* = 1.5E-7), and 0.39 (*p* = 1.9E-11), for all KDO8PSs and for the C23 and N23 subfamilies, respectively, suggesting that the choice of sequence in the regions of high relative entropy is at least in part aimed at increasing the overall stability of the structure. If the correlation vectors between sequence and stability for each sub-family (3^rd^ and 4^th^ insets of Figure 2B) are subtracted from each other (5th inset of Figure 2B), a new set of positions is identified in the structure of *Nm.* KDO8PS, which represent places in which the correlation with stability changes significantly between the two sub-families. These are positions in which one sub-family selectively affects stability with respect to the other sub-family by preferentially adopting or discarding certain amino acids. By and large, these positions (Figure 1B, residues colored in magenta) are also found outside the β-barrel, as already noted for the positions derived from the difference between the HMM vectors (Figure 1B, residues colored in green).

### The contribution of coevolving residues to the stability of KDO8PS

In a MSA some positions are highly conserved, while others vary. The conserved positions are clearly important, but the non-conserved positions are not irrelevant because the net stabilization of the folded state, relative to the unfolded state is usually so small, that all positions may contribute significantly to the protein stability. This is clearly evident in the stability landscape of Figure 2A. Thus, the destabilizing effects of a given amino acid at one position can be compensated by the stabilizing effect of a certain amino acid at another position: in other words, two positions could be coevolving. A wide variety of algorithms have been developed to detect coevolving positions from a MSA (reviewed in [40], [41], [42]). Some of these methods use 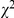-tests [43], [44], some are perturbative [45], [46], [47], others employ amino acid substitution matrices [48], and many work within the frame of information theory [49]. Information entropy, *H*(X), is a measure of the uncertainty associated with a discrete random variable X that assumes values {x_1_,..., x_n_}:
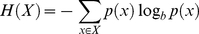
(2)where *b* is the base of the logarithm used and *p* is the probability mass function of the variable X [50], [51]. Related to *H*(X), mutual information, *MI*(X;Y), measures the mutual dependence of two discrete random variables X and Y:
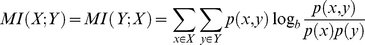
(3)where *p*(x,y) is the joint probability mass function of X and Y, and *p*(x) and *p*(y) are the marginal probability mass functions of X and Y, respectively. Intuitively, MI measures how much knowing one of the two variables reduces the uncertainty about the other. In a MSA, the amino acids in a given column can be considered as a set of observations (x_i_) of a random variable X. An estimate of the entropy *H*(X) is obtained by using the observed amino acid frequencies, *f*(x_i_), in place of the underlying probabilities, *p*(x_i_); likewise, *MI*(X;Y) for a pair of columns can be derived using the frequencies, *f*(x_i_,y_j_), of all ordered pairs occurring in the two columns. In practice, MI between positions (columns in a MSA) reflects the extent to which knowledge of the amino acid at one position allows us to predict the identity of the amino acid at the other position [52], [53], [54]. If amino acids occur independently at the two sites, the theoretical value for MI is zero; conversely, MI is high if the two positions are correlated.

However, significant background MI can originate from random pairings of residues when the number of sequences in the multiple sequence alignments is small (in practice, less than 125 sequences [54]). In addition, positions with high entropy (non-conserved positions) have more background MI than positions with low entropy. MI is also affected by various sources of bias, because the sequences in a MSA do not exactly meet the assumption of independent evolution. For example, the appearance of a mutation in an ancestral protein, which is clearly a single evolutionary event, would be considered in a MI analysis as representing an independent event that occurred in each of the proteins in the MSA that descended from that ancestor. This treatment of a single event as multiple independent events acts as a phylogenetic bias that increases the mutual information among residues. Normalization of MI values reduces the effect of positional entropy and phylogenetic bias [54], and several normalized variants of MI have been proposed. A useful and symmetric type of normalized MI is symmetric uncertainty (*SU*) [55], [56]:
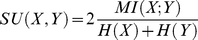
(4)


*MIr*, a form of MI normalized by the joint entropy of the variables, *H*(X,Y), instead of the sum, *H*(X)+*H*(Y), is conceptually similar to SU and is also widely used [53], [54].

We have calculated the matrix of symmetric uncertainties *SU*(X,Y) for all columns in the MSAs of the entire family of metallo + non-metallo KDO8PSs. As recommended by some authors [57], [58], [59] gaps were not included in the amino acid alphabet as this can lead to artificially high MI values, and if a gap appeared in a row in at least one of the two columns, that row did not contribute to the SU value for just those two columns. On the other hand, also this exclusion may lead to an artificial increase of the SU value. In fact, while the theoretical value for MI is zero if amino acids occur independently at the two sites, MI can be zero only if the observed pair frequencies reflect all possible pairs for the observed amino acid frequencies. If all 20 amino acids are present in each column and are equally probable, MI vanishes only if the frequency of each pair of amino acids is 1/20^2^. This condition is not met in a MSA with less than 400 sequences. Thus, in order to weigh the significance of pairs of columns containing different numbers of ungapped rows, the SU value was scaled linearly by the fraction of ungapped rows in the two columns with respect to the total number of rows in the alignment. Other forms of (non-linear) correction for the number of rows included in the MI calculation have also been proposed [60].

Many have noticed that MI between positions *i* and *j* in a MSA is highly correlated to the product 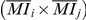 of the average value of MI at each of these positions [58], [61]. While the origin of this correlation is uncertain, and can perhaps be attributed to positional entropy effects and a combination of both phylogenetic and stochastic bias [61], it is nonetheless clear that it produces a high background MI that obscures coevolution patterns. Several corrections have been proposed to eliminate this correlation, giving rise to new formulations or scoring of MI defined respectively as positional MI, *MIp* [58], Z-scored residual MI, *ZRes* [61], Z-scored-product normalized MI, *ZNMI* [62]. The *Zpx* score introduced by Gloor *et al.* [63] supercedes the *MIp* score. More recently it has been pointed out that attempts to infer coevolution only from pair-count data are heavily affected by assumptions on the consistency between joint and marginal frequencies [59], and that additional biological knowledge may be necessary for a meaningful derivation of coevolution patterns. In this line of thinking, Codoner *et al.* [64] have proposed to consider the correlation in the hydrophobicity and/or molecular weight of coevolving amino acid sites *a priori* to determine statistically their biological significance, and have shown that the application of these statistical filters to the number of pairs detected as coevolving reduces significantly the number of false positives. However, while this is clearly an emerging strategy in coevolution studies, here we are primarily interested in determining whether there is a statistical correlation between coevolution and stability, and therefore we do not include in the identification of coevolving pairs any statistical filters based on structural properties that directly affect stability (like hydrophobicity or molecular weight).

Finally, the sensitivity of MI analyses to the size and quality of the MSA is also a matter of concern [60], [62], [64], [65]. We have studied the effect of different levels of sequence redundancy in the MSA, by calculating MI not only with the original data set of 348 sequences but also with a series of progressively smaller MSAs in which the highest level of identity between any two sequences was 98, 96, 94, 92, 90, 88, 86, 84, 82, 80%. These MSAs consisted respectively of 308, 266, 241, 221, 203, 179, 165, 154, 146, 130 sequences. A threshold point at which the trend in the statistics for the total number and distribution of coevolving pairs appeared to change could be recognized in the MSA with 86% maximal sequence identity, which consisted of only 165 sequences (Figure S2, MSA S3, Supporting Information). While throughout the manuscript we refer to the complete data set of 348 sequences, the corresponding results obtained with the data set of 165 sequences are also provided (Table S1, Table S2, Table S3, Supporting Information). Overall these analyses were fairly insensitive to the size/redundancy of the MSAs, suggesting that in the specific case of KDO8PS MI studies are not particularly affected by stochastic and/or phylogenetic bias.

**Figure 3:**
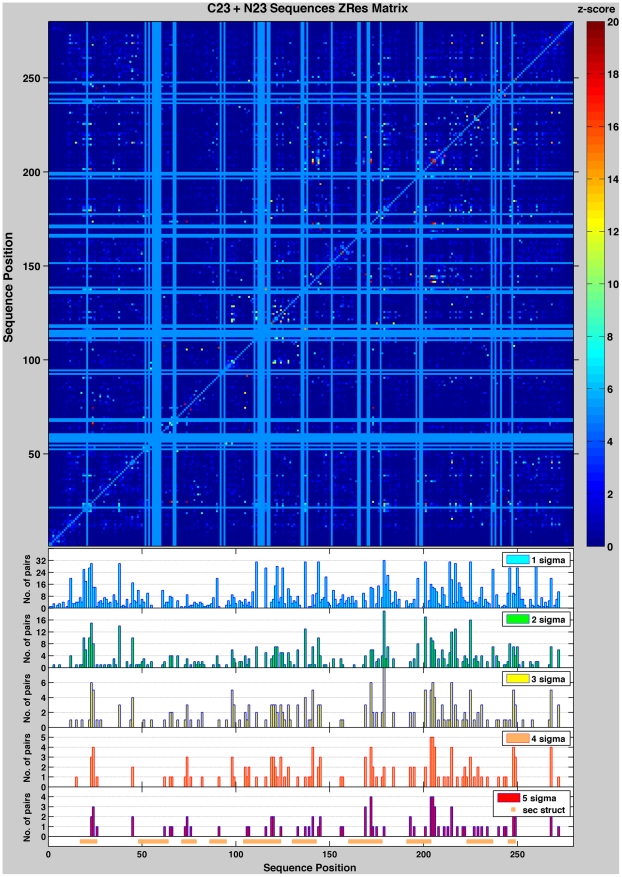
Mutual information in KDO8PS. The *ZRes* matrix for the MSA of all KDO8PSs is shown in the upper panel. For better contrast, matrix values are displayed in the z-score range 0 to 20 with the color ramp shown in the side bar; the full range of the matrix is from −3.9 to 245.7. Fully conserved positions appear as uniform light blue rows and columns. For each position of the MSA of all KDO8PSs, the insets below the matrix show the number of coevolving pairs with score values larger 1, 2, 3, 4, or 5 σ over the mean of all the scores in the matrix. The regions of the structure with the highest relative entropy in the HMM vector of Figure 1A are shown as orange bars below the histograms; these are the same regions also shown as orange bars in Figures 1 and 2.

Despite normalization and scaling, the SU matrix of the MSA for the KDO8PS family is contaminated by a significant level of background MI (*corr* = 0.78 to the 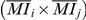 column product matrix). The *Zpx*, *ZRes* and *ZNMI* matrices all work well with the KDO8PS data set in reducing this background MI; as expected they are highly correlated to each other (*corr*(*Zpx,ZRes*) = 0.90, *corr*(*Zpx,ZNMI*) = 0.81, *corr*(*ZNMI,ZRes*) = 0.73). Since it is not clear yet which of these formulations of MI is more accurate or appropriate for a particular study [62], we report the results obtained with each one in our analysis of coevolution in KDO8PS (Tables 1, 2 and 3). In this study we were not interested in determining whether there are patterns of spatial relationship among the coevolving positions, but whether there is a relationship between coevolving pairs and the stability landscape of KDO8PS described in the previous section. As an example of our approach, the number of coevolving pairs with score higher than 1 to 5 σ over the mean of all scores in the *ZRes* matrix, are shown for every position in the MSA of all KDO8PSs as histograms in the insets of Figure 3. The same regions of sequence highlighted for the HMM vector of Figure 1A are shown below the baseline of the lowest inset, as horizontal orange bars. The correlation coefficients between the histogram vectors for each type of MI matrix and the HMM vector of Figure 1A are shown in Table 1. These coefficients were calculated including only positions of the MSA that do not correspond to fully conserved residues, as there are no coevolving pairs between these positions. Some modest level of correlation (at best *corr* = 0.30) with reasonable statistical significance (*p*<0.01) can be recognized only for the strongest signals in the *ZRes*, and *ZNMI* matrices.

**Table 1:** Correlation coefficients between the histogram vectors derived from the MI matrices and the HMM vector of Figure 1A.

Threshold for coevolving pairs	1 σ	2 σ	3 σ	4 σ	5 σ
	Correlation to the HMM vector [*p-*value]				
***Zpx*** ** matrix**	−0.393 [2.0e-10]	−0.094 [0.144]	0.061 [0.346]	0.104 [0.012]	0.118 [8.0e-4]
***ZRes*** ** matrix**	−0.013 [0.838]	0.118 [0.066]	0.171 [0.007]	0.222 [4.9e-4]	0.201 [1.6e-3]
***ZNMI*** ** matrix**	−0.270 [1.9e-5]	0.104 [0.105]	0.227 [3.4e-4]	0.297 [2.3e-6]	0.300 [1.7e-6]

**Table 2:** Contribution to KDO8PS stability from coevolving pairs with score >5 σ in the *ZRes* matrix of Figure 3.

*i,j pair*	score	ΔΔG(i+j)	|ΔΔG(i-j)|	*i,j pair*	score	ΔΔG(i+j)	|ΔΔG(i-j)|
23,179	13.464	1.473	0.866	145,169	18.448	0.481	0.299
23,201	16.307	1.264	0.674	**145,193**	**13.953**	**0.380**	**0.399**
24,65	20.772	0.494	0.302	156,157	23.782	1.169	0.428
24,66	16.083	0.600	0.203	169,193	26.494	0.217	0.209
24,74	19.011	0.594	0.217	169,218	14.159	0.686	0.553
**26,244**	**245.65**	**−0.021**	**0.024**	172,204	20.79	0.294	0.205
45,215	13.477	0.602	0.505	172,205	16.916	0.516	0.424
45,268	13.995	1.112	0.651	172,206	15.603	0.413	0.333
**62,107**	**188.1**	**−0.009**	**0.030**	195,221	71.347	0.285	0.252
**73,76**	**18.029**	**0.174**	**0.387**	204,205	24.25	0.709	0.235
74,173	18.367	0.378	0.285	204,206	19.581	0.606	0.356
**91,106**	**16.741**	**0.272**	**0.323**	205,206	16.747	0.828	0.579
116,137	14.649	2.573	2.497	208,211	15.733	0.326	0.274
119,120	41.6	0.025	0.025	215,249	14.89	0.176	0.081
119,175	13.509	0.094	0.083	215,268	14.422	0.662	0.518
120,124	13.63	0.073	0.058	**222,248**	**14.622**	**0.241**	**0.256**
133,234	114.09	0.111	0.092	227,229	14.026	1.276	0.971
**139,213**	**184.35**	**−0.067**	**0.083**	231,268	13.469	2.913	1.800
141,172	17.902	0.194	0.136	240,243	15.497	0.872	0.805
141,204	17.453	0.388	0.120	**248,249**	**45.125**	**0.162**	**0.186**
144,205	13.579	0.782	0.160	268,272	28.615	0.820	0.440

ΔΔG's are in kcal/mol. Rows in which |ΔΔG(i-j)|>ΔΔG(i+j) are shown in bold.

**Table 3:** Correlation coefficients between the vectors of MI scores for *i,j* pairs above a threshold σ value and the vectors representing the average effect of those pairs on the stability of KDO8PS.

Threshold for coevolving pairs.	1 σ	2 σ	3 σ	4 σ	5 σ
	***Zpx*** ** matrix**				
**No. of unique coevolving pairs** a	4319	1402	467	162	75
***corr*** **(MI_ij_,ΔΔG_i_+ΔΔG_j_)** [*p*-value]^b^	−0.019 [0.103]	−0.110 [1.9e-5]	−0.205 [3.8e-6]	−0.272 [2.3e-4]	−0.258 [0.013]
***corr*** **(MI_ij_,|ΔΔG_i_−ΔΔG_j_|)** [*p*-value]	−0.082 [3.0e-8]	−0.156 [2.0e-9]	−0.223 [5.5e-7]	−0.283 [1.3e-4]	−0.260 [0.013]
**% of pairs with opposite effects**	0.343	0.301	0.244	0.222	0.223
	***ZRes*** ** matrix**				
**No. of unique coevolving pairs**	861	267	105	66	42
***corr*** **(MI_ij_,ΔΔG_i_+ΔΔG_j_)** [*p*-value]	−0.116 [3.3e-4]	−0.183 [1.3e-3]	−0.236 [7.7e-3]	−0.260 [0.017]	−0.353 [0.011]
***corr*** **(MI_ij_,|ΔΔG_i_–ΔΔG_j_|)** [*p*-value]	−0.129 [7.1e-5]	−0.194 [7.3e-4]	−0.231 [9.0e-3]	−0.240 [0.026]	−0.288 [0.032]
**% of pairs with opposite effects**	0.271	0.232	0.238	0.227	0.190
	***ZNMI*** ** matrix**				
**No. of unique coevolving pairs**	4157	892	225	78	32
***corr*** **(MI_ij_,ΔΔG_i_+ΔΔG_j_)** [*p*-value]	−0.051 [4.5e-4]	−0.166 [3.3e-7]	−0.242 [1.2e-4]	−0.235 [0.019]	−0.451 [4.8e-3]
***corr*** **(MI_ij_,|ΔΔG_i_−ΔΔG_j_|)** [*p*-value]	−0.105 [5.5e-12]	−0.201 [7.1e-10]	−0.262 [3.5e-5]	−0.238 [0.018]	−0.445 [5.3e-3]
**% of pairs with opposite effects**	0.362	0.314	0.280	0.269	0.250

a Since the MI matrix is symmetric the total number of pairs (as represented for example in the histograms of Figure 3) is twice that of the unique part of the matrix.

b The *null* hypothesis of zero correlation was tested against the *alternative* hypothesis of negative correlation.

Since the HMM vector represents deviations from the expected background probability distribution to satisfy the demands of structure, stability and function, it is reasonable to suggest that strongly coevolving positions of KDO8PS may be associated with one or more of these demands. In order to determine the specific contribution of coevolution to stability we compared the level of MI of each pair with the average contribution to stability by that pair in the MSA of KDO8PS, as derived by applying to each sequence in the MSA the information contained in the stability matrix shown in Figure 2A (Energy Matrix S1, Supporting Information). For example, based on that matrix a proline would contribute 3.27 kcal/mol at position 31 of a sequence in the MSA, but −1.05 kcal/mol at position 56. We were interested not only in the sum (ΔΔG*_i_*+ΔΔG*_j_*) of the stability contributions of each member of the *i,j* pair (as these contributions can be considered approximately additive under the assumption of no epistasis), but also in the absolute value of the difference |ΔΔG*_i_*−ΔΔG*_j_*|, which depends on whether the two contributions have similar or opposite effects. Since MI is already the log of a probability, consistent with equation (1) the two magnitudes of interest are:
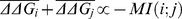
(5)

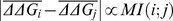
(6)where the average in equation (6) is over all the sequences in the MSA. As an example, these magnitudes are shown in Table 2 for all coevolving pairs with a score >5 σ in the *ZRes* matrix of Figure 3. Less than 20% of these pairs (rows in bold in Table 2) have values of |ΔΔG*_i_*−ΔΔG*_j_*|>(ΔΔG*_i_*+ΔΔG*_j_*), and thus identify positions that, on average, exert opposite effects on the stability of KDO8PS. For each type of MI matrix, the correlations between the score vector (for example, the score columns in Table 2) and the (ΔΔG*_i_*+ΔΔG*_j_*) and |ΔΔG*_i_*−ΔΔG*_j_*| vectors are shown in Table 3. In general, it can be seen that, as progressively higher σ thresholds are chosen, the score vectors become more clearly anti-correlated to the (ΔΔG*_i_*+ΔΔG*_j_*) vector: this trend is consistent with equation (5), because we expect that pairs whose components raise the ΔΔG are less likely to occur. The score vectors are also anti-correlated to the |ΔΔG*_i_*−ΔΔG*_j_*| vector, suggesting that on average coevolving pairs have a preference for positions whose individual effects on stability are similar. The trends are similar in the various matrices and *p*-values are acceptable, with *ZRes* and *ZNMI* yielding the largest values of anti-correlation (Table 3).

These results can be interpreted in the light of current theories of coevolution. According to one hypothesis, a reason for coevolving pairs is to suppress a decrease in stability or function produced by a mutation at one site with an increase in stability or function provided by a mutation in a residue near the site of the first mutation [66], [67], [68]. This tenet is challenged by the observation that most suppressor mutations are not close in space to the initial mutations [69], while coevolving sites are most often spatially clustered [53], [54], [68], [70]. Directed evolution studies also suggest that epistatic paths can be bypassed (and they certainly are in laboratory evolution), because there are multiple sequences that satisfy a given fitness goal, and there are many different paths to these sequences [34], [71], [72].

According to an alternative model for coevolution, “covarions” arise when both the original residue and the mutated residue are compatible with function, but the spectrum of residues possible at other positions in the protein is altered by the mutation [73]. In this context one might expect that most single mutations that finally become stabilized in coevolving pairs are neutral in the context of the protein in which they occur, but become beneficial (even at a much later time) in the presence of the partner in the pair [63]. The data in Table 3 suggest that both mechanisms were operational in the evolution of KDO8PS. Approximately 1/4 of all strongly coevolving pairs (for example those shown in bold in Table 2; see also Table 3) may have originated from cycles of mutation and suppression that affected stability. Other pairs, for which both values of (ΔΔG*_i_*+ΔΔG*_j_*) and |ΔΔG*_i_*−ΔΔG*_j_*| are small, are best explained by a succession of neutral or nearly neutral covarions.

## Discussion

In the metal dependent forms of KDO8PS the metal is not directly involved in an activation process, but together with its ligands stabilizes the position of the reactants that favors the condensation reaction [7], [13], [14]. In the non-metal forms of the enzyme, this role is performed by an asparagine side chain that replaces the combination cysteine + metal [13], [14]. In both forms of the enzyme additional stabilization of the reactants is provided by variations in the CysAspGlyPro motif of the loop that contains the metal binding aspartic acid [6], [12]. Outside these demands placed on the structure by the need to catalyze a specific reaction, the factors (like protein stability) that contributed to the evolution of hundreds of different forms of KDO8PS are not well understood. The starting point of our study was the derivation of the stability landscape of KDO8PS (Figure 2A) by calculating the ΔΔG changes associated with introducing any one of the 20 possible amino acids at each sequence position of the structure of *Nm.* KDO8PS (taken here as the reference structure for this class of proteins). Superposition on this landscape, of the actual amino acid frequencies at each position in the two sub-families of KDO8PSs confirms the intuitive expectation that in most positions of high relative entropy (Figure 1A, 1^st^ inset), the amino acids most often used are those that do not decrease stability (Figure 2B, 1^st^ inset). Furthermore, the plane regions of the landscape tend to be large, such that random drifts of sequence have a high chance of producing only small gains or losses of stability, which can be easily offset by changes in other regions. This feature of the landscape may be part of the physical basis for the threshold robustness (tolerance) to mutations that was observed in other proteins [74], [75].

Another important factor in constraining sequence variation is the need to retain a common core structure, while possibly adapting to specific bacterial environments. Ultimately, the preservation of structure is related to the preservation of function because small changes in atom distances in the active site (produced by overall changes of structure) can have effects on the activation energies as dramatic as those produced by very localized mutations in the active sites [76]. Both sequence conservation and coevolution were involved in the preservation of the structure of KDO8PS, but coevolution had a marginal role (Table 1). This result can be rationalized by observing that if most mutations shifted a position in the sequence away from a clearly defined valley in the stability landscape, then it would be more likely for a given amino acid at that position to become fully conserved. Conversely, if most mutations moved a position around between regions of similar height, a large number of different amino acids at other positions might easily compensate the small ΔΔG changes of the initial mutation, and a clear pattern of coevolution would be less likely to emerge.

In our study we have used various formulations of MI to identify correlated positions, but other methods may provide different information on the potential cross-talks between conserved and correlated positions, and be better suited for different tasks [44]. For example, using the Observed Minus Expected Squared (OMES) covariance method [43] Kowarsch *et al.* found a statistically significant association between correlated positions and disease-causing mutations [77]. The association was more pronounced for the exposed accessible sites of the proteins studied, which are expected to be more involved with function, rather than for the structural cores, which are more likely to be involved in stability. Thus, altogether, despite the significantly different algorithm used, the conclusions of Kowarsch's study are compatible and complementary to ours in assessing the contributions of correlated positions to the stability of proteins.

Derivation of the stability landscape is still at an early stage of refinement and its utility as a tool to understand the evolution of amino acid sequences in protein families needs additional confirmations. Current calculations were carried out with the FoldX algorithm, which uses a full atomic description of the structure of proteins and whose different energy terms have been weighted using empirical data obtained from protein engineering experiments [22], [29], [78]. While this or similar algorithms involving empirical functions provide a relatively fast estimation of the importance of the interactions contributing to the stability of proteins and protein complexes, the calculated ΔΔG changes are unlikely to be on a correct absolute scale [30] and are certainly not as accurate as those derived from more sophisticated approaches like, for example, Free Energy Perturbation (FEP [79]); unfortunately, FEP is still computationally too expensive and time consuming, to be compatible with full sequence scans. Two recent comparisons of various methods (including CC/PBSA [80], Rosetta [81], EGAD [82], I-Mutant2.0 [83], I-Mutant3.0 [84], CUPSAT [85], MultiMutate [86], Dmutant [87], Hunter [31], and FoldX) designed to predict the ΔΔG changes associated with mutations, ranked FoldX among the overall best performing ones [31], [32]. However, while all the methods showed a correct trend in their predictions, they failed to provide precise values, with the best predictors being only moderately (60%) accurate.

All these considerations point to the conclusion that significantly better tools are needed for a quantitative analysis of mutations by computational means. However, regardless of whether further improvements are easily achievable in calculating the stability landscape of proteins, an aim of this study is to stress how such landscape can be an important tool to integrate structural data with information theory to understand the evolution of proteins. For example, it should be noted that HMM profiles would be quite different if the information content of the distribution *P*_i_ at the *i*-th position of the profile was calculated with respect to the expected frequency of different amino acids as derived from the values of the stability matrix at that position, rather than from the background frequencies of different amino acids in all known proteins, or just in the family under consideration. In that case we might expect that the positions with high information content would be those in which the observed sequence deviates significantly (perhaps for functional reasons) from the stability constraints associated with a certain structure. Similar considerations might apply also to the calculation of mutual information, where the expected frequencies derived from the stability profile could be used as the probability mass function of each variable. This approach is complementary to that adopted by Codoner *et al.* [64] to filter out (based on the hydrophobicity and molecular weight of residues at specific positions) potential false positives in the ensemble of coevolving pairs inferred from MI analyses. For example, if a model based on the size of side chains is adopted for the distribution of aa's at any given position, we can see how the *largest* contribution to MI between any two positions will originate from deviations (like the match of one large and one small side chain in a defined region of space) of the observed joint distribution with respect to the model. The interpretation of MI as the Kullback-Leibler divergence [88], [89], [90] of the observed *versus* the model joint distribution of two variables becomes particularly meaningful in this context.

While our study was primarily aimed at studying the effect of coevolving residues on the overall stability of KDO8PS, indirectly it provided important information on the functional role of specific pairs. For example, residues 23–26 of *Nm.* KDO8PS listed at the top of Table 2 represent the sequence AsnValLeuGlu, with Asn being the key residue the replaces the metal binding Cys in all non-metal KDO8PS's. Position 26 appears to coevolve with position 244, which immediately precedes the conserved motif CysAspGlyPro (residues 246–249 in *Nm.* KDO8PS), whose functional role in KDO8PS was already noted [12]. Coevolution of position 24 with positions 65,66, and 74 is explained by the fact that residue 24 is in van der Waals contact with 66 and within 10 Å of 65 and 74. Other pairs, like position 23 (Cys or Asn in all KDO8PS) with positions 179 and 201, are of interest but at the moment still unexplained. It is also worth noting that, besides its relevance for the coevolution analysis, the stability matrix of *Nm.* KDO8PS (Energy Matrix S1, Supporting Information) provides the platform for designing new mutations of KDO8PS that may be useful to stabilize or destabilize specific regions of the enzyme.

Finally, it is legitimate to ask whether the conclusions about the role of coevolving residues in the thermodynamic stability of KDO8PS can be extended to other proteins. First, the analysis appears to be fairly insensitive to the choice of the reference sequence in the MSA. In fact, we obtained very similar results using the X-ray structure of KDO8PS from the hyperthermophile *Aquifex aeolicus* (PDB entry 1FWW) as the reference structure for the calculation of the stability matrix (not shown). Second, preliminary application of the method to two other structurally unrelated protein families (the Atp12p chaperones involved in F_1_-ATPase assembly [91], and B1 type metallo β-lactamases [92]) gave results that are consistent with those obtained with KDO8PS (Table S4, Table S5, Supporting Information). While these observations are very encouraging, clearly more testing with a larger set of different protein families will be necessary before any general principles about the role of coevolution in protein stability can be derived from the application of the tools described in this study.

## Methods

### Multiple Sequence Alignments

Multiple sequence alignments (MSAs) of 348 sequences (175 metallo and 173 non-metallo KDO8PSs) were calculated independently with T-Coffee [93], Muscle [94], and Mafft [95] and then merged together with T-Coffee (MSA S1, Supporting Information). For clarity and ease of comparison with the X-ray structure, the sequence numbering of *Nm.* KDO8PS was used as reference for the entire family, and only positions in the MSA with a corresponding residue in *Nm.* KDO8PS were finally retained (MSA S2, Supporting Information). Hidden Markov Models (HMMs) [25] were calculated with the HMMER 3.0 package [96].

### Molecular Dynamics Simulations

A complete three-dimensional model of *Nm.* KDO8PS was built with Prime 2.1 (Schrodinger, LLC) using primarily the X-ray structure of *Nm.* KDO8PS (PDB 2QKF) as template, and that of *Aa.* KDO8PS (PDB 1FWW) only to build the residues missing in the *Nm.* structure. The ensemble for the MD simulation was constructed with Desmond (D.E. Shaw Research) [97] by solvating the enzyme with SPC water [98] inside an orthorhombic box of 87.7, 110.0, 105.4 Å: a minimum distance of 12 Å was left between any protein atom and the edge of the box. The final ensemble contained a tetramer of *Nm.* KDO8PS, approximately 26,000 solvent molecules, 72 Na and 72 Cl ions in the solvent regions to neutralize charges on the proteins and to achieve a final salt concentration of 150 mM. Prior to additional steps the ensemble was subjected to energy minimization under periodic boundaries condition with a totals of 2,000 iterations first with the steepest descent (SD) method until a gradient threshold of 25 kcal/mol/Å was achieved, and then with the LBFGS method [99] until a convergence threshold of 1 kcal/mol/Å was met. The 2005 release of the OPLS-AA force-field [100] was used in this and all subsequent calculations. Short range Coulombic interactions were calculated with a cutoff radius of 9.0 Å, while long range interactions were calculated with the smooth particle mesh Ewald method [101] using an Ewald tolerance of 1e^−9^. A 12 nanoseconds (ns) MD simulation of the solvated tetrameric *Nm.* KDO8PS was carried out with Desmond in the NPT ensemble at 300 K. For this purpose the Nose-Hoover thermostat method [102] with a relaxation time of 1.0 picoseconds (ps), and the Martyna-Tobias-Klein barostat method [103] with isotropic coupling of the cell along all three axes to a reference pressure of 1.01325 atm and a relaxation time of 2 ps were used. Integration was carried out with the RESPA integrator [104] using time steps of 2.0 fentomseconds (fs), 2.0 fs, and 6.0 fs for the bonded, van der Waals and short-range, and long-range electrostatic interactions. SHAKE constraints [105] were imposed on all the heavy-atom-hydrogen covalent bonds. Coulombic interactions were calculated as for the minimization protocol. Coordinates were saved every 4.8 ps. Before the 12 ns productive run of the simulation, the ensemble was relaxed using the following protocol: 1) 12 ps in the NVT ensemble at 10 K with a fast relaxation constant and non-hydrogen solute atoms restrained; 2) 12 ps in the NPT ensemble at 10 K and 1 atm, with a fast temperature relaxation constant, a slow pressure relaxation constant, and non-hydrogen solute atoms restrained; 3) 24 ps at 300 K and 1 atm with other conditions as in step 2; 4) 24 ps at 300 K and 1 atm with a fast temperature relaxation constant and a fast pressure relaxation constant.

Conformational clustering based on the cross-RMSDs between the frames contained in the 6–12 ns interval was carried out with the program X-Cluster (Schrodinger, LLC).

### FoldX calculations of protein stability

ΔΔG changes associated with introducing any one of the 20 possible amino acids at each position in all four monomers of a tetramer (the biological unit) of *Nm.* KDO8PS were calculated with FoldX v3.0b4 [22], [29] following the procedure described in [106]. First, each of the representative structures of *Nm.* KDO8PS derived from the MD simulation was optimized using the repair function of FoldX. Then, structures corresponding to each of the point mutants were generated and their energies calculated using the FoldX energy function. Finally, the energy of the optimized wild-type structure was subtracted from that of the mutant. Each calculation was carried out in duplicate to ensure convergence: in this case the FoldX algorithm repeated the same mutations twice changing the rotamer set used and the order of moves such that alternative solutions could be explored.

### Mutual Information Analysis

Correlation coefficients were calculated with the Statistics Toolbox of Matlab 7.10 (The MathWorks™). Mutual Information (MI) and Symmetric Uncertainty (SU) were calculated with the Information Theory Toolbox v.1.0 for Matlab developed by Joaquin Goni (Dept. of Physics and Applied Mathematics, University of Navarra, Pamplona, Spain), and available for download at Matlabcentral (www.mathworks.com/matlabcentral). Z-scored cross-product positional MI, *Zpx* [63], product based Z-scored residual MI, *ZRes* [61], Z-scored-product normalized MI, *ZNMI* [62] were implemented according to the published algorithms as Matlab programs. All the data sets and Matlab programs required to reproduce the results of the study are available from the authors upon request. Figures were generated with Matlab 7.10 and Pymol 1.2r3 (Schrodinger, LLC).

## Supporting Information

Figure S1 **Derivation of the stability landscape of** ***Nm.*** **KDO8PS.** A. MD simulation of tetrameric *Nm.* KDO8PS at 300K. The Cα root mean square deviation, Cα-RMSD, from the structure at time *t* = 0 is shown in the upper trace colored in blue (0–6 ns) and red (6–12 ns). The fluctuations around the average structure (Cα root mean square fluctuation, Cα-RMSF) that occur during the 6–12 ns part of the simulation are shown in the lower green trace, and reflect the degree of mobility in the solution structure. B. ΔΔG changes associated with mutating every amino acid of all four subunits of *Nm.* KDO8PS to all 20 possible amino acids (a total of 5600 mutations in each subunit) were calculated in duplicate for each of the three main conformers (PDB S1, PDB S2, PDB S3) observed in the 6–12 ns part of the MD simulation. Values derived from these three representative configurations were then merged (magenta trace; see also Energy Matrix S1) according to their contribution (relative ratio of 58∶24∶18 for PDB S1∶PDB S2∶PDB S3) to the solution ensemble. Standard deviations of the ΔΔG values are shown as a black trace.
(TIF)

Figure S2 **Effect of redundancy in the MSA of KDO8PSs on the identification of coevolving pairs.** A. MI with *ZRes* scoring was calculated for the original data set of 348 sequences (maximum 99% identity between any two sequences) and for a series of smaller MSAs in which the highest level of identity between any two sequences was 98, 96, 94, 92, 90, 88, 86, 84, 82, 80%. The total number of coevolving positions (left panel) or unique coevolving pairs (right panel) is shown for different σ levels in the MI matrix. B. The level of contrast in the MI matrix is expressed as the ratio between the number of coevolving positions (left panel) or unique coevolving pairs (right panel) at 5 and 1 σ, respectively.
(TIF)

Table S1 Correlation coefficients between the histogram vectors derived from the MI matrices and the HMM vector of a MSA of 165 KDO8PS sequences (MSA S3) in which the highest identity allowed between any two sequences is 86%.
(DOC)

Table S2 Contribution to KDO8PS stability from coevolving pairs with score >5 σ in the *ZRes* matrix of a MSA of 165 KDO8PS sequences (MSA S3) in which the highest identity allowed between any two sequences is 86%. ΔΔG's are in kcal/mol. Rows in which |ΔΔG(i−j)|>ΔΔG(i+j) are shown in bold.
(DOC)

Table S3 Correlation coefficients between the vectors of MI scores for *i,j* pairs above a threshold σ value and the vectors representing the average effect of those pairs on the stability of KDO8PS, based on a MSA of 165 KDO8PS sequences (MSA S3) in which the highest identity allowed between any two sequences is 86%.
(DOC)

MSA S1 MSA in fasta format of all KDO8P synthases.
(FASTA)

MSA S2 MSA in fasta format of all KDO8P synthases with only the positions corresponding to the sequence of *Nm.* KDO8PS (Uniref entry 9JZ55) retained.
(FASTA)

MSA S3 MSA in fasta format of KDO8P synthases with no more than 86% identity between any two sequences. Only the positions corresponding to the sequence of *Nm.* KDO8PS (Uniref entry 9JZ55) were retained.
(FASTA)

Table S4 Correlation coefficients between the vectors of MI scores (based on a MSA of 249 sequences) for *i,j* pairs above a threshold σ value and the vectors representing the average effect of those pairs on the stability of Atp12p.
(DOC)

Table S5 Correlation coefficients between the vectors of MI scores (based on a MSA of 145 sequences) for *i,j* pairs above a threshold σ value and the vectors representing the average effect of those pairs on the stability of B1 type metallo β-lactamases.
(DOC)

PDB S1 Atomic coordinates in pdb format for the structure of *Nm.* KDO8PS which, based on a clustering procedure, is the most representative conformer of all the states sampled during the 6–12 ns part of the MD run shown in Figure S1.
(PDB)

PDB S2 Atomic coordinates in pdb format for the structure of *Nm.* KDO8PS which, based on a clustering procedure, is the 2^nd^ most representative conformer of all the states sampled during the 6–12 ns part of the MD run shown in Figure S1.
(PDB)

PDB S3 Atomic coordinates in pdb format for the structure of *Nm.* KDO8PS which, based on a clustering procedure, is the 3^rd^ most representative conformer of all the states sampled during the 6–12 ns part of the MD run shown in Figure S1.
(PDB)

Energy Matrix S1 FoldX energy matrix. The rows (1–280, top to bottom) of the matrix correspond to the amino acid sequence of *Nm.* KDO8PS. The columns (1–20, left to right) of the matrix correspond to the 20 common amino acids in the following order: G A L V I P R T S C M K E Q D N W Y F H. ΔΔG values are in kcal/mol.
(TXT)
