# Supplementary figures and images for: The Contribution of Coevolving Residues to the Stability of KDO8P Synthase
Source: PLoS One. 2011 Mar 9;6(3):e17459. doi: 10.1371/journal.pone.0017459 (PMC3052366; doi:10.1371/journal.pone.0017459)

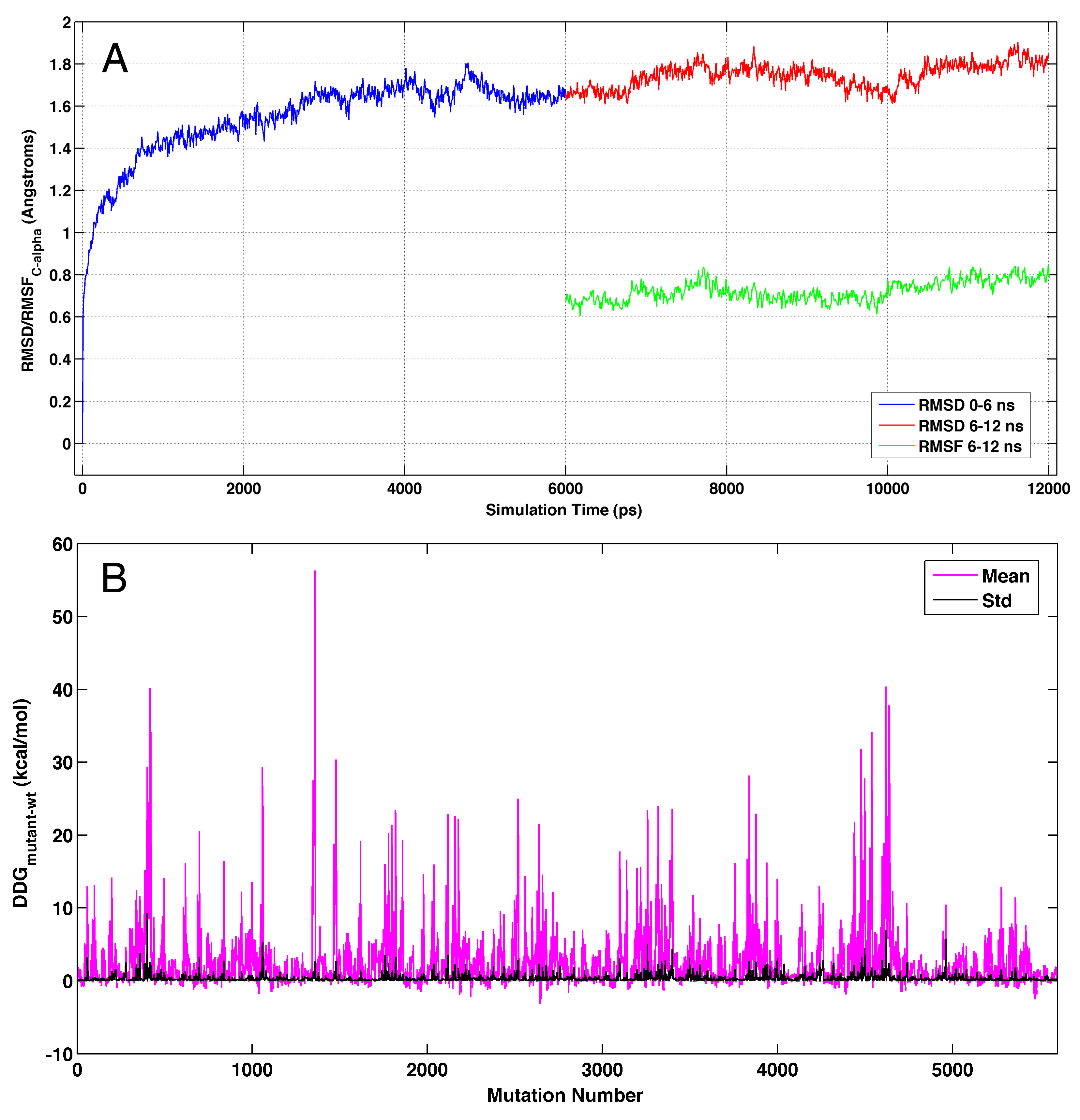

Supplement: Figure S1 — Derivation of the stability landscape of Nm. KDO8PS. A. MD simulation of tetrameric Nm. KDO8PS at 300K. The Cα root mean square deviation, Cα-RMSD, from the structure at time t = 0 is shown in the upper trace colored in blue (0–6 ns) and red (6–12 ns). The fluctuations around the average structure (Cα root mean square fluctuation, Cα-RMSF) that occur during the 6–12 ns part of the simulation are shown in the lower green trace, and reflect the degree of mobility in the solution structure. B. ΔΔG changes associated with mutating every amino acid of all four subunits of Nm. KDO8PS to all 20 possible amino acids (a total of 5600 mutations in each subunit) were calculated in duplicate for each of the three main conformers (PDB S1, PDB S2, PDB S3) observed in the 6–12 ns part of the MD simulation. Values derived from these three representative configurations were then merged (magenta trace; see also Energy Matrix S1) according to their contribution (relative ratio of 58∶24∶18 for PDB S1∶PDB S2∶PDB S3) to the solution ensemble. Standard deviations of the ΔΔG values are shown as a black trace. (TIF) [file pone.0017459.s001.tif]

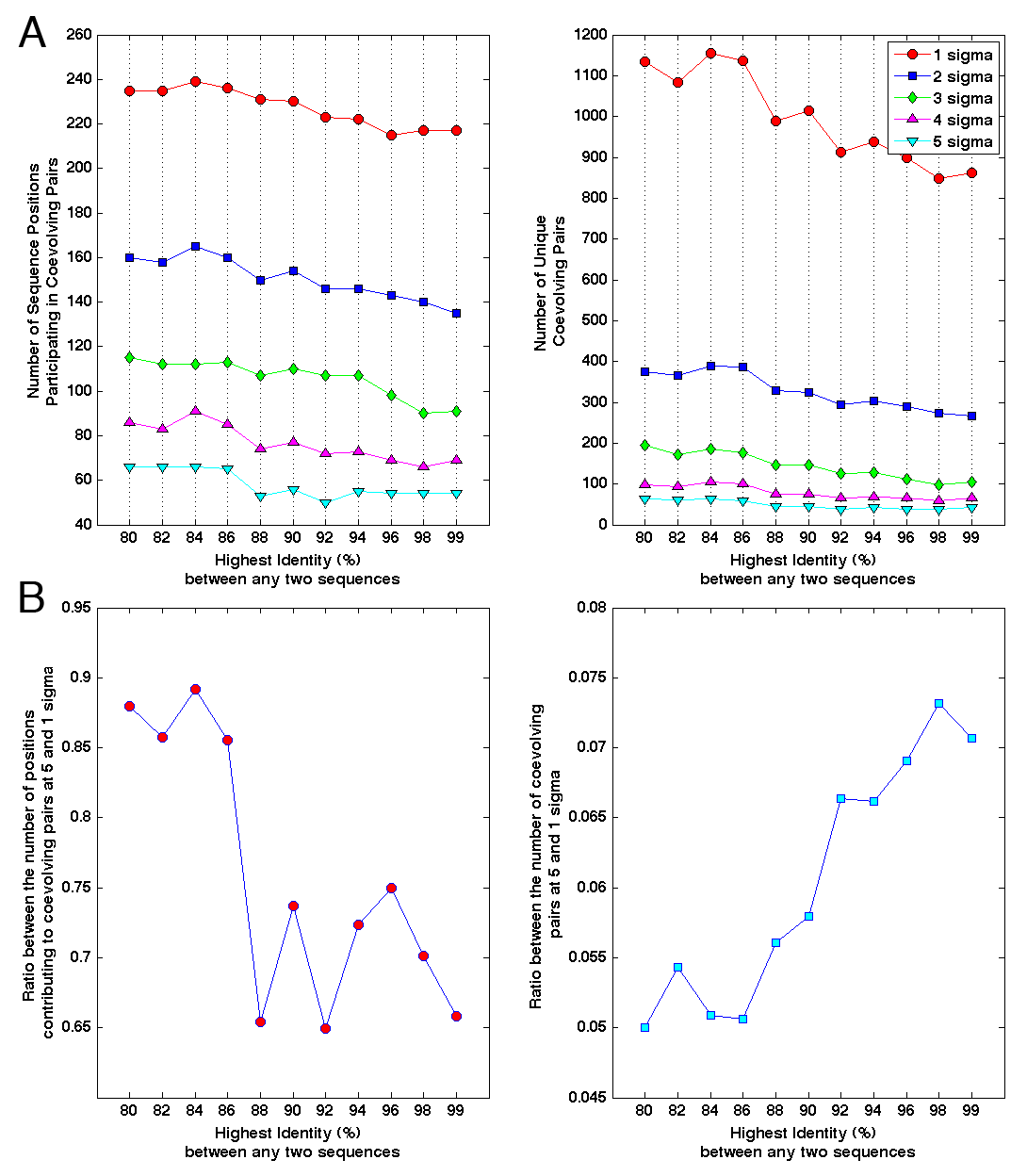

Supplement: Figure S2 — Effect of redundancy in the MSA of KDO8PSs on the identification of coevolving pairs. A. MI with ZRes scoring was calculated for the original data set of 348 sequences (maximum 99% identity between any two sequences) and for a series of smaller MSAs in which the highest level of identity between any two sequences was 98, 96, 94, 92, 90, 88, 86, 84, 82, 80%. The total number of coevolving positions (left panel) or unique coevolving pairs (right panel) is shown for different σ levels in the MI matrix. B. The level of contrast in the MI matrix is expressed as the ratio between the number of coevolving positions (left panel) or unique coevolving pairs (right panel) at 5 and 1 σ, respectively. (TIF) [file pone.0017459.s002.tif]
