# Supplementary material for: The Contribution of Coevolving Residues to the Stability of KDO8P Synthase
Source: PLoS One. 2011 Mar 9;6(3):e17459. doi: 10.1371/journal.pone.0017459 (PMC3052366; doi:10.1371/journal.pone.0017459)
Supplement: Table S1 — Correlation coefficients between the histogram vectors derived from the MI matrices and the HMM vector of a MSA of 165 KDO8PS sequences (MSA S3) in which the highest identity allowed between any two sequences is 86%. (DOC) [file pone.0017459.s003.doc]

**Table S1.** Correlation coefficients between the histogram vectors derived from the MI matrices and the HMM vector (based on a MSA of 165 KDO8PS sequences in which the highest identity allowed between any two sequences is 86%).

| **Threshold for**  **coevolving pairs** | **1 σ** | **2 σ** | **3 σ** | **4 σ** | **5 σ** |
| --- | --- | --- | --- | --- | --- |
|  | **Correlation to the HMM vector** [*p-*value] | | | | |
| ***Zpx* matrix** | -0.473 [5.5e-15] | -0.144 [0.024] | 0.064 [0.318] | 0.138 [0.031] | 0.163 [0.011] |
| ***ZRes* matrix** | -0.084 [0.192] | 0.087 [0.176] | 0.140 [0.028] | 0.154 [0.016] | 0.175 [0.006] |
| ***ZNMI* matrix** | -0.347 [2.6e-8] | 0.076 [0.238] | 0.198 [0.002] | 0.252 [6.7e-5] | 0.285 [6.1e-6] |
