# Supplementary material for: The Contribution of Coevolving Residues to the Stability of KDO8P Synthase
Source: PLoS One. 2011 Mar 9;6(3):e17459. doi: 10.1371/journal.pone.0017459 (PMC3052366; doi:10.1371/journal.pone.0017459)
Supplement: Table S2 — Contribution to KDO8PS stability from coevolving pairs with score >5 σ in the ZRes matrix of a MSA of 165 KDO8PS sequences (MSA S3) in which the highest identity allowed between any two sequences is 86%. ΔΔG's are in kcal/mol. Rows in which |ΔΔG(i−j)|>ΔΔG(i+j) are shown in bold. (DOC) [file pone.0017459.s004.doc]

**Table S2.** Contribution to KDO8PS stability from coevolving pairs with score > 5 σ in the *ZRes* matrix (based on a MSA of 165 KDO8PS sequences in which the highest identity allowed between any two sequences is 86%). ΔΔG’s are in kcal/mol. Rows in which |ΔΔG(i-j)| > ΔΔG(i+j) are shown in bold.

| ***i,j pair*** | **score** | **ΔΔG(i+j)** | **|ΔΔG(i-j)|** |  | ***i,j pair*** | **score** | **ΔΔG(i+j)** | **|ΔΔG(i-j)|** |
| --- | --- | --- | --- | --- | --- | --- | --- | --- |
| 12,231 | 11.677 | 3.488 | 2.926 |  | 133,223 | 10.730 | 2.402 | 2.302 |
| 18,133 | 12.464 | 0.412 | 0.349 |  | **133,234** | **33.184** | **0.072** | **0.115** |
| 22,179 | 12.108 | 3.990 | 1.240 |  | **133,248** | **31.465** | **0.181** | **0.196** |
| 23,179 | 18.066 | 1.760 | 1.027 |  | 137,179 | 12.128 | 4.349 | 1.571 |
| 23,201 | 17.867 | 1.546 | 0.827 |  | **139,213** | **143.38** | **-0.047** | **0.100** |
| **23,217** | **11.016** | **0.021** | **0.755** |  | 141,172 | 16.938 | 0.234 | 0.157 |
| 23,225 | 10.774 | 0.515 | 0.350 |  | 141,204 | 18.656 | 0.441 | 0.134 |
| 24,65 | 21.017 | 0.621 | 0.365 |  | 141,206 | 11.656 | 0.467 | 0.239 |
| 24,66 | 20.049 | 0.733 | 0.253 |  | **142,224** | **14.088** | **0.123** | **0.215** |
| 24,74 | 16.908 | 0.712 | 0.288 |  | 144,204 | 12.999 | 0.641 | 0.179 |
| 24,173 | 11.951 | 0.674 | 0.442 |  | 144,205 | 14.512 | 0.900 | 0.169 |
| **26,244** | **207.15** | **-0.020** | **0.024** |  | 156,157 | 25.941 | 1.128 | 0.409 |
| 28,80 | 14.446 | 2.353 | 1.384 |  | 172,204 | 19.651 | 0.335 | 0.231 |
| 45,253 | 11.429 | 1.684 | 0.876 |  | 172,205 | 16.471 | 0.593 | 0.480 |
| **62,107** | **120.09** | **-0.003** | **0.032** |  | 172,206 | 14.939 | 0.361 | 0.272 |
| 62,133 | 24.992 | 0.0953 | 0.091 |  | 179,225 | 12.18 | 1.543 | 1.326 |
| 65,66 | 11.388 | 0.426 | 0.164 |  | **192,248** | **13.777** | **0.168** | **0.259** |
| **73,76** | **13.937** | **0.274** | **0.460** |  | 195,221 | 53.019 | 0.490 | 0.387 |
| 74,173 | 10.796 | 0.459 | 0.351 |  | 197,201 | 11.033 | 2.922 | 0.612 |
| 88,215 | 16.628 | 1.247 | 1.135 |  | 201,225 | 11.768 | 1.320 | 1.144 |
| **91,106** | **32.153** | **0.316** | **0.396** |  | 204,205 | 25.34 | 0.800 | 0.268 |
| 98,120 | 19.826 | 0.277 | 0.240 |  | 204,206 | 19.133 | 0.567 | 0.301 |
| 98,124 | 16.339 | 0.311 | 0.245 |  | 205,206 | 17.258 | 0.826 | 0.544 |
| **107,133** | **29.822** | **0.067** | **0.102** |  | 208,211 | 12.39 | 0.329 | 0.263 |
| **107,248** | **25.684** | **0.0821** | **0.202** |  | **211,212** | **14.904** | **0.389** | **0.562** |
| **111,122** | **13.601** | **1.535** | **2.201** |  | 227,229 | 11.742 | 1.270 | 0.965 |
| 111,125 | 11.317 | 4.660 | 1.011 |  | 231,268 | 11.988 | 3.942 | 2.421 |
| 116,137 | 16.036 | 2.993 | 2.922 |  | **248,249** | **48.89** | **0.215** | **0.226** |
| 120,124 | 28.049 | 0.0835 | 0.058 |  | 268,272 | 26.722 | 1.196 | 0.584 |
| 133,145 | 11.875 | 0.439 | 0.415 |  |  |  |  |  |
