# Supplementary material for: The Contribution of Coevolving Residues to the Stability of KDO8P Synthase
Source: PLoS One. 2011 Mar 9;6(3):e17459. doi: 10.1371/journal.pone.0017459 (PMC3052366; doi:10.1371/journal.pone.0017459)
Supplement: Table S3 — Correlation coefficients between the vectors of MI scores for i,j pairs above a threshold σ value and the vectors representing the average effect of those pairs on the stability of KDO8PS, based on a MSA of 165 KDO8PS sequences (MSA S3) in which the highest identity allowed between any two sequences is 86%. (DOC) [file pone.0017459.s005.doc]

**Table S3.** Correlation coefficients between the vectors of MI scores for *i,j* pairs above a threshold σ value and the vectors representing the average effect of those pairs on the stability of KDO8PS (based on a MSA of 165 KDO8PS sequences in which the highest identity allowed between any two sequences is 86%).

| **Threshold for coevolving pairs** | **1 σ** | **2 σ** | **3 σ** | **4 σ** | **5 σ** |
| --- | --- | --- | --- | --- | --- |
|  | ***Zpx* matrix** | | | | |
| **No. of unique coevolving pairs** | 4321 | 1408 | 478 | 184 | 73 |
| ***corr*(MI_ij_,ΔΔG_i_ + ΔΔG_j_)** [*p*-value]^a^ | 0.011 [0.775] | -0.087 [05.6e-4] | -0.160 [2.3e-4] | -0.240 [5.2e-4] | -0.337 [1.8e-3] |
| ***corr*(MI_ij_,|ΔΔG_i_ - ΔΔG_j_|)** [*p*-value] | -0.059 [5.7e-5] | -0.152 [4.6e-9] | -0.201 [4.9e-6] | -0.260 [1.8e-4] | -0.323 [2.7e-3] |
| **% of pairs with opposite effects** | 0.340 | 0.283 | 0.241 | 0.228 | 0.233 |
|  | ***ZRes* matrix** | | | | |
| **No. of unique coevolving pairs** | 1137 | 386 | 176 | 100 | 59 |
| ***corr*(MI_ij_,ΔΔG_i_ + ΔΔG_j_)** [*p*-value] | -0.094 [7.7e-4] | -0.152 [1.3e-3] | -0.206 [3.1e-3] | -0.267 [3.6e-3] | -0.282 [0.015] |
| ***corr*(MI_ij_,|ΔΔG_i_ - ΔΔG_j_|)** [*p*-value] | -0.122 [1.9e-05] | -0.162 [6.85e-4] | -0.220 [1.7e-3] | -0.261 [4.4e-3] | -0.269 [0.020] |
| **% of pairs with opposite effects** | 0.268 | 0.228 | 0.222 | 0.22 | 0.254 |
|  | ***ZNMI* matrix** | | | | |
| **No. of unique coevolving pairs** | 4200 | 884 | 241 | 73 | 37 |
| ***corr*(MI_ij_,ΔΔG_i_ + ΔΔG_j_)** [*p*-value] | -0.010 [0.262] | -0.148 [5.0e-6] | -0.225 [2.1e-4] | -0.338 [1.7e-3] | -0.370 [0.012] |
| ***corr*(MI_ij_,|ΔΔG_i_ - ΔΔG_j_|)** [*p*-value] | -0.078 [2.0e-7] | -0.193 [3.8e-9] | -0.243 [7.1e-5] | -0.337 [1.8e-3] | -0.292 [0.040] |
| **% of pairs with opposite effects** | 0.360 | 0.281 | 0.266 | 0.274 | 0.378 |

**^a^**The *null* hypothesis of zero correlation was tested against the *alternative* hypothesis of negative correlation.
