# Supplementary material for: The Contribution of Coevolving Residues to the Stability of KDO8P Synthase
Source: PLoS One. 2011 Mar 9;6(3):e17459. doi: 10.1371/journal.pone.0017459 (PMC3052366; doi:10.1371/journal.pone.0017459)
Supplement: Table S4 — Correlation coefficients between the vectors of MI scores (based on a MSA of 249 sequences) for i,j pairs above a threshold σ value and the vectors representing the average effect of those pairs on the stability of Atp12p. (DOC) [file pone.0017459.s009.doc]

**Table S4.** Correlation coefficients between the vectors of MI scores (based on the MSA of 249 sequences) for *i,j* pairs above a threshold σ value and the vectors representing the average effect of those pairs on the stability of Atp12p.

| **Threshold for coevolving pairs** | **1 σ** | | **2 σ** | | **3 σ** | | **4 σ** | | **5 σ** | |
| --- | --- | --- | --- | --- | --- | --- | --- | --- | --- | --- |
|  | ***Zpx* matrix** | | | | | | | | | |
| **No. of unique coevolving pairs** | 4.200 |  | 1.300 |  | 399 |  | 156 |  | 67 |  |
| ***corr*(MI_ij_,ΔΔG_i_ + ΔΔG_j_)** [*p*-value]**^a^** | -0.056 | 1.6E-4 | -0.066 | 0.007 | -0.104 | 0.019 | -0.164 | 0.020 | -0.288 | 0.009 |
| ***corr*(MI_ij_,|ΔΔG_i_ - ΔΔG_j_|)** [*p*-value] | -0.106 | 4E-12 | -0.167 | 4E-10 | -0.255 | 1.2E-7 | -0.31 | 4.0E-5 | -0.385 | 6.4E-4 |
| **% of pairs with opposite effects** | 0.564 |  | 0.553 |  | 0.489 |  | 0.404 |  | 0.358 |  |
|  | ***ZRes* matrix** | | | | | | | | | |
| **No. of unique coevolving pairs** | 1600 |  | 435 |  | 195 |  | 110 |  | 68 |  |
| ***corr*(MI_ij_,ΔΔG_i_ + ΔΔG_j_)** [*p*-value] | -0.066 | 0.004 | -0.108 | 0.0122 | -0.19 | 0.004 | -0.252 | 0.004 | -0.286 | 0.009 |
| ***corr*(MI_ij_,|ΔΔG_i_ - ΔΔG_j_|)** [*p*-value] | -0.145 | 3.0E-9 | -0.239 | 2.4E-7 | -0.301 | 9.7E-6 | -0.371 | 3.3E-5 | -0.373 | 8.6E-4 |
| **% of pairs with opposite effects** | 0.557 |  | 0.506 |  | 0.421 |  | 0.373 | 0 | 0.368 |  |
|  | ***ZNMI* matrix** | | | | | | | | | |
| **No. of unique coevolving pairs** | 4003 |  | 484 |  | 98 |  | 34 |  | 22 |  |
| ***corr*(MI_ij_,ΔΔG_i_ + ΔΔG_j_)** [*p*-value] | -0.058 | 1.33E-4 | -0.138 | 0.001 | -0.283 | 0.002 | -0.296 | 0.045 | -0.396 | 0.034 |
| ***corr*(MI_ij_,|ΔΔG_i_ - ΔΔG_j_|)** [*p*-value] | -0.113 | 5.5E-13 | -0.268 | 1.0E-9 | -0.377 | 6.6E-5 | -0.305 | 0.040 | -0.371 | 0.045 |
| **% of pairs with opposite effects** | 0.561 |  | 0.469 |  | 0.357 |  | 0.294 |  | 0.318 |  |

**^a^**The *null* hypothesis of zero correlation was tested against the *alternative* hypothesis of negative correlation.
