# Supplementary material for: The Contribution of Coevolving Residues to the Stability of KDO8P Synthase
Source: PLoS One. 2011 Mar 9;6(3):e17459. doi: 10.1371/journal.pone.0017459 (PMC3052366; doi:10.1371/journal.pone.0017459)
Supplement: Table S5 — Correlation coefficients between the vectors of MI scores (based on a MSA of 145 sequences) for i,j pairs above a threshold σ value and the vectors representing the average effect of those pairs on the stability of B1 type metallo β-lactamases. (DOC) [file pone.0017459.s010.doc]

**Table S5.** Correlation coefficients between the vectors of MI scores (based on the MSA of 145 sequences) for *i,j* pairs above a threshold σ value and the vectors representing the average effect of those pairs on the stability of B1 type metallo β-lactamases.

| **Threshold for coevolving pairs** | **1 σ** | | **2 σ** | | **3 σ** | | **4 σ** | | **5 σ** | |
| --- | --- | --- | --- | --- | --- | --- | --- | --- | --- | --- |
|  | ***Zpx* matrix** | | | | | | | | | |
| **No. of unique coevolving pairs** | 4700 |  | 997 |  | 237 |  | 81 |  | 40 |  |
| ***corr*(MI_ij_,ΔΔG_i_ + ΔΔG_j_)** [*p*-value]**^a^** | 0.024 | 0.946 | -0.073 | 0.011 | -0.182 | 2.5E-3 | -0.315 | 2.1E-3 | -0.294 | 0.033 |
| ***corr*(MI_ij_,|ΔΔG_i_ - ΔΔG_j_|)** [*p*-value] | -0.014 | 0.181 | -0.111 | 2.4E-4 | -0.204 | 7.9E-4 | -0.289 | 4.4E-3 | -0.312 | 0.025 |
| **% of pairs with opposite effects** | 0.519 |  | 0.474 |  | 0.439 |  | 0.407 |  | 0.3 |  |
|  | ***ZRes* matrix** | | | | | | | | | |
| **No. of unique coevolving pairs** | 999 |  | 219 |  | 86 |  | 50 |  | 36 |  |
| ***corr*(MI_ij_,ΔΔG_i_ + ΔΔG_j_)** [*p*-value] | -0.075 | 0.009 | -0.162 | 0.008 | -0.259 | 0.008 | -0.289 | 0.021 | -0.28 | 0.049 |
| ***corr*(MI_ij_,|ΔΔG_i_ - ΔΔG_j_|)** [*p*-value] | -0.105 | 4.8E-4 | -0.173 | 0.005 | -0.222 | 0.020 | -0.274 | 0.027 | -0.244 | 0.075 |
| **% of pairs with opposite effects** | 0.473 |  | 0.447 |  | 0.384 |  | 0.36 |  | 0.278 |  |
|  | ***ZNMI* matrix** | | | | | | | | | |
| **No. of unique coevolving pairs** | 3860 |  | 345 |  | 80 |  | 29 |  | 18 |  |
| ***corr*(MI_ij_,ΔΔG_i_ + ΔΔG_j_)** [*p*-value] | 0.008 | 0.692 | -0.143 | 0.004 | -0.327 | 1.6E-3 | -0.31 | 0.051 | -0.198 | 0.215 |
| ***corr*(MI_ij_,|ΔΔG_i_ - ΔΔG_j_|)** [*p*-value] | -0.029 | 0.039 | -0.178 | 4.5E-4 | -0.3 | 3.4E-3 | -0.283 | 0.069 | -0.203 | 0.209 |
| **% of pairs with opposite effects** | 0.508 |  | 0.426 |  | 0.375 | 0 | 0.172 |  | 0.278 |  |

**^a^**The *null* hypothesis of zero correlation was tested against the *alternative* hypothesis of negative correlation.
